# Work–Life Balance: A Different Scale for Doctors

**DOI:** 10.3389/fped.2015.00115

**Published:** 2015-12-24

**Authors:** I. Federico Fernandez Nievas, Danyal Thaver

**Affiliations:** ^1^Division of Critical Care Medicine, Department of Pediatrics, Golisano Children’s Hospital, Upstate University, Syracuse, NY, USA

**Keywords:** work–life balance, residency, pediatric intensive care unit, workload, job satisfaction, burnout, professional

## The Resident Perspective

“Hey, do you want to hang out this weekend?”

“Ummm … let me see if I’m on call …”

This is a conversation every resident goes through almost every month of their training. Despite work hour regulations, overnight calls and weekend calls define and shape our life. Every family event, birthday, and anniversary has to be adjusted around the work schedule. Weekends are traditionally seen as family time. Not so much during residency. At the start of residency, every intern is promised that with work hour regulations now in effect (1), more time can be spent at home with family. Little do they realize that they make up for the reduced duty hours by working more weekends. Even with compensatory time, family time is unlikely to be recovered (2).

I got married just before starting my residency. At the time, I did not realize how much the residency would come in the way of my personal life. However, on my days off, I found myself catching up on lost sleep and regaining the strength to survive another day. Just like most other chronically sleep-deprived residents (3), even when I woke up, I was grumpy and irritable (4). Let us just say my wife was not amused.

Starting the second year of residency was a scary thought not just in terms of increased responsibility, but also because it posed the physical challenge of working for 24 h straight. The Accreditation Council for Graduate Medical Education (ACGME) encourages residents to take a “strategic nap” during these shifts, which made me think it should not be as bad as I thought. And it was not. A 60–90-min nap seemed sufficient to keep me going for 24 h. However, during my pediatric intensive care unit (PICU) rotation, the same strategy did not work. “Strategic naps” were now only 10 min cat naps on a chair. Like with any ICU rotation, there were highs and lows. Although it was a roller-coaster ride, I thoroughly enjoyed the rotation. The challenge of taking care of the sickest children in the hospital and the immediate gratification of curing someone kept me going.

While I was engrossed in the sustained chaos in the ICU, I was disconnected from the outside world. For those 4 weeks, I was solely driven by my passion to take care of acutely sick patients. No matter how physically or mentally tired I might have been, those patients were more precious than my sleep. I soon realized that this chronic lack of sleep and the mental and emotional fatigue made me neglect my family and affected my mood. Any other concerns that my friends or family had seemed less important to me and did not warrant immediate attention compared to acutely ill children holding on to their lives. This made me question my priorities. Is my triage of responsibilities right? Is my work schedule affecting my attitude toward non-urgent concerns outside the hospital? And most importantly, do I want to do this for the rest of my life?

During residency, the academic *curriculum* is very well outlined. However, not as much time is spent teaching residents the importance of striking a balance, or at least trying, between their stressful and long work hours and their personal life. Most Graduate Medical Education (GME) offices do offer services to residents that may include sports facilities and corporate discounts, that can be helpful. Many more stress management strategies can be learnt from experienced physicians that may be formally incorporated into medical training (5). Sometimes, the vague transition from work to home can be eased by conscious physical detachment from work. Google ran a program in its Dublin office called “Dublin Goes Dark” where employees were not allowed to take their electronic devices home and had to leave them at work. The forced detachment from work led to more stress-free evenings (6). Many residents struggle not to use home access to electronic medical records to check on how some patients are doing since their last shift or check the clinical census before they start the next shift. Formal training on making sure that we do not remain preoccupied about a child’s suffering once we go back home is vital.

I have learned this the hard way. After a couple of weeks in the PICU, I wanted to emotionally blind myself from the hospital once I was done with my shift and going back home. This may sound inhumane and insensitive. However, I could not spend the few hours or a rare weekend that I had off, obsessed with something I could do nothing about. No matter how much I wanted to, I could not be in the hospital 24/7. This was my time. And I had to claim it.

It worked! Time spent outside of work started becoming more satisfying and enjoyable. I felt more refreshed and less emotionally exhausted. There was still one major problem: time. With more than half of the time in a week spent working, I felt that I was still neglecting family duties and furthermore neglecting myself. Time at home was spent studying, preparing for presentations, and working on research projects. Essentially, still work. I used to paint and go to the gym regularly. Not anymore. I complained to myself and my family complained to me. I could not prolong my week and get an extra day in the week and I felt helpless.

I pondered over this dilemma and realized that the problem lay within my own expectations. Becoming a doctor came with a price and I should be ready to pay it. Family life of a doctor is a different “normal.” The sooner I accepted this and lowered my expectations, the sooner would I be more satisfied with the “balance” that I was trying to achieve. I started utilizing my time wisely. At work, I would squeeze reading assignments into lunch breaks and tried to work on projects while still in the hospital. At home, I had to plan weekends, holidays, and free evenings. Exercise could not be an hour long session, it could still be 15 min. Slowly, my wife started coming to terms with the limited time we could spend together. We made the most of that quality time that was otherwise not maximized. We decided to plan each weekend more than we had ever done.

From medical school to residency to fellowship and eventually full-time work, life is only going to get more demanding. I did not want to believe this, but now I do. Caring for acutely sick children is what I love to do the most. Accepting that this is going to be challenging and demanding in many ways only helps motivate me and realize that achieving work–life balance will be a constant struggle. As long as I know that my work may help a child reach his potential, I am willing to give up weekends and accept sleepless nights.

“Happiness can only exist in acceptance” – George Orwell

## The Attending Physician Perspective

From the moment, we decide to study medicine and work as physicians, we need to realize that work will absorb a large part of our life, where long hours and weekend calls are just the tip of the iceberg. Along with the need to study and continuously update our knowledge from the current literature, as physicians, we will have many other career demands, such as Board exams, Maintenance of Certification, and GME requirements. The sooner we understand this reality and accept it, the better. Spending time worrying about how much we are working or how many calls we have to do would make our job twice as hard, we are getting stressed thinking about it and we still have to do the work. Do not get me wrong, I am not advocating we should accept any unfair work conditions or do not try to improve our quality of life, but living continuously stressed and dissatisfied will drain further our already depleted energy. This vicious cycle could lead to burn out, depression, addiction, isolation, family problems including divorce. A study found higher prevalence for burn out in physicians compared to other workers in the United States and a higher risk among primary care physicians (7). The increased burden of paperwork, electronic medical records, and the pressure to see more patients are making physicians leave clinical practice prematurely and switch to a different career path or give up completely medicine to pursue better work conditions in an entirely different field. When an experienced physician quits practice, it is very difficult and costly to replace him. Additionally, there are data predicting a physician shortage in the next 5–10 years, since the projected increase in physician number will not match the projected growth demand (8).

There is an increased focus on the millennial generation of physicians since it is believed that this group has more expectations about work hours, flexibility, benefits, and the importance of work–life balance. The millennials are described as the “wanting all” generation (9). Consequently, efforts must be taken to improve job satisfaction for the physicians, especially for those from the Millennial generation, who are going to play an increasingly central role in the Health Care work force as they replace the soon to retire the Baby Boomer doctors (10, 11).

Difficult work environment and inefficient leaders are also important contributors to job dissatisfaction besides the long working hours and patient overload. Thus, when searching for jobs, attention should be paid to a broad spectrum of work conditions, and work satisfaction should be considered a priority, not a mere side effect. This alone will, in the long run, optimize performance and grant better work–life balance, and ultimately, maximize career achievements.

In my opinion, considering work and life as two opposite forces is like dividing ourselves into two pieces that cannot coexist. We are one and we should not isolate the physician from the person. Each of these aspects of our life contributes to who we are, to our happiness, and ultimately to our success as physicians and human beings.

## Author Contributions

Each author wrote a part of this article. IN also edited and formatted. Both authors contributed equally and are first authors of this article.

## Conflict of Interest Statement

The authors declare that the research was conducted in the absence of any commercial or financial relationships that could be construed as a potential conflict of interest.
